# Comparison of Vitamin D Level of Children with Severe Early Childhood Caries and Children with No Caries

**DOI:** 10.5005/jp-journals-10005-1511

**Published:** 2018-06-01

**Authors:** Anchal Chhonkar, Anil Gupta, Vishal Arya

**Affiliations:** 1 Postgraduate Student, Department of Pedodontics and Preventive Dentistry, Shree Guru Gobind Singh Tricentenary Dental College Hospital & Research Institute, New Delhi, India; 2 Head, Department of Pedodontics and Preventive Dentistry, Shree Guru Gobind Singh Tricentenary University, Gurugram, Haryana India; 3 Professor, Department of Pedodontics and Preventive Dentistry, Shree Guru Gobind Singh Tricentenary University, Gurugram, Haryana India

**Keywords:** Case-control study, Severe early childhood caries, Vitamin D deficiency.

## Abstract

**Aim:**

To compare the levels of vitamin D in children with severe early childhood caries (SECC) and children without caries and to determine the association of vitamin D deficiency and SECC.

**Materials and methods:**

A total of 30 children each from case (with caries) and control group (without caries) of age between 3 and 6 years were selected from the Department of Pedo-dontics and Preventive Dentistry, Faculty of Dental Sciences, SGT University, Gurugram. Caries status of the children was recorded using decayed, extracted, and filled teeth (deft) index. Blood samples for serum 25(OH) vitamin D were taken from each child. All the data collected were compiled and subjected to appropriate statistical analysis.

**Results:**

Case group has mean serum 25(OH) vitamin D level of 12.19 ng/mL [4.37 standard deviation (SD), 95% confidence interval of 10.5-13.8] and control group has mean serum 25(OH) vitamin D level of 20.11 ng/mL (4.12 SD, 95% confidence interval of 18.56-21.65). When the mean levels of serum 25(OH) vitamin D were compared between case and control groups, there was a statistically significant difference (p-value <0.0001). Simple linear regression in case group shows statistically significant inverse correlation between vitamin D levels and SECC (p-value<0.0001).

**Conclusion:**

Our results showed that vitamin D deficiency is risk factor both for incidence of dental caries and for its severity in children.

**Clinical significance:**

Vitamin D deficiency is an important modifiable risk factor for dental caries in children. Hence, by supplementing vitamin D in children and preventing the deficiency of vitamin D, dental caries can be prevented.

**How to cite this article:** Chhonkar A, Gupta A, Arya V. Comparison of Vitamin D Level of Children with Severe Early Childhood Caries and Children with No Caries. Int J Clin Pediatr Dent 2018;11(3):199-204.

## INTRODUCTION

Early childhood caries is defined as the presence of one or more decayed (noncavitated or cavitated lesions), missing (due to caries), or filled tooth surfaces in any primary tooth in a child 71 months of age or younger.^1^

Early childhood caries is a considerable public health problem. In rural areas in India, 80% of the children and 60% of the adults suffer from dental caries.^2-4^ It is the most common prevalent chronic infectious childhood disease, and it is difficult to control and can start as soon as the infant’s teeth erupt.^5-7^ Multiple factors play a role in the development of dental decay, including microorganisms, diet, oral hygiene, medical conditions, and lack of important nutrients, such as vitamin D.^7,8^ Worldwide, 1 billion people are at risk of vitamin D deficiency^9,10^ and vitamin D plays a crucial role in the absorption of calcium and phosphorus.

The interaction of 1,25-dihydroxyvitamin D with the vitamin D receptor increases the efficiency of absorption of intestinal calcium by up to 40% and phosphorus up to 80%.^11,12^ Vitamin D deficiency is defined as a 25-hydroxyvitamin D (25[OH] D) level of less than 20 ng/mL.^13,14^ There are many causes of vitamin D deficiency, including heritable disorders like obesity,^15^ dark skin^16,17^ and acquired disorders like lack of sunlight, drugs,^18^ and malabsorption. Higher serum levels of 25-hydroxy vitamin D [25(OH)D] above 30 nm/mL are associated with improved oral health outcomes.^19^ Vitamin D also shows an immunological role, as it can induce the formation of antimicrobial peptides, such as cathelicidin and defensins.

Salivary antimicrobial peptides concentrations showed large differences between individuals, with a significantly higher level of salivary defensins in children with no caries.^20,21^ The secretion rate and quality of the saliva are important in caries development and also in remineralization. Saliva is key factor to maintain the integrity of the teeth as well as the soft tissues of oral cavity.^22^ Despite the fact that vitamin D deficiency and SECC are common conditions worldwide, very few studies have been done to establish the correlation between vitamin D deficiency and SECC.^23-26^ Even after extensive literature search, there is no such study done in India to determine the association between vitamin D deficiency and SECC.

Hence, there is a need for such studies in India. With this background, this study was done with the objective to compare the levels of vitamin D in children with SECC and caries-free children and to determine the association of vitamin D deficiency and SECC.

## MATERIALS AND METHODS

This case-control study was conducted in the Department of Pedodontics and Preventive Dentistry, in collaboration with the Department of Pediatrics, SGT University, Budhera, Gurugram, Harayana, India. Two groups of 30 children each were included in the study, and were classified under the case group and the control group; the distribution of these groups was as follows.

The case group: This group comprised of 30 children aged between 3 and 6 years with multiple decayed teeth who were selected from the Outpatient Department of Pedodontics and Preventive Dentistry, SGT Dental College, Bhudera, Gurugram, Harayana, India. The control group: The control group comprised of 30 children aged between 3 and 6 years with no caries teeth who were selected from the Outpatient Department of Pediatrics, SGT Hospital, Budhera, Gurugram, Harayana, India.

Inclusion criteria for the case group: 1. Healthy children between 3 and 6 years of age with no chronic medical illness. 2. Presence of SECC pathology.

Inclusion criteria for the control group: 1. Healthy children between 3 and 6 years of age with no chronic medical illness. 2. Patients who present with no frank cavitated lesions upon visual examination (deft 0). Since all patients were selected at SGT University, they all belonged to the same socioeconomic status. For each child of case and control groups, following data were collected.

Recording of dental caries status: Oral examination of each child was done to record the total number of deft. Examination of all the children in both the groups was conducted by one investigator using proper light and with the help of probe/explorer and mouth mirror after proper drying of the teeth. Caries status of the children was recorded using deft index.

The dental caries status for both the groups was noted in a proforma attached. A designed questionnaire was prepared to determine the child’s medical history, oral health, eating habits, parent’s oral health, and socioeconomic status, and was filled by parents of children of both the case and the control groups. A venous blood sample (approximately 2.5 mL) was obtained after taking consent from the parents, by a technician from the clinical pathology laboratory for the estimation of serum 25(OH) vitamin D. All the data collected were compiled and subjected to statistical analysis using mean, paired t-test, and simple linear regression, and compilation of the result was done.

## RESULTS

A total of 60 children were recruited and divided into two groups of 30 children with SECC (case group) and 30 children with no caries (control group). Comparison of the demographic profile of case and control groups was done. Both groups showed no difference in terms of age and male/female ratio (Table 1). Age, sex, deft score, and serum 25(OH) vitamin D levels in the case group were collected.

**Graph 1:**
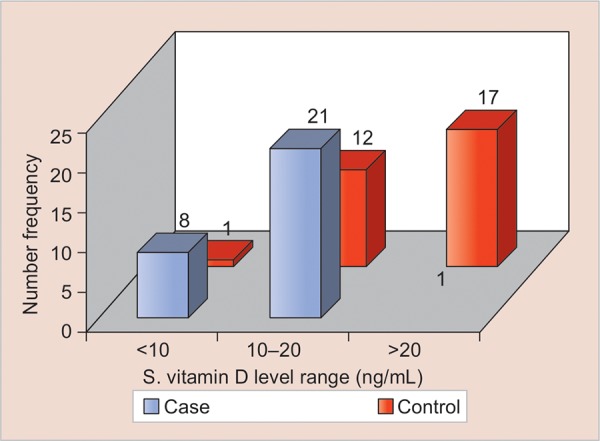
Number of children in case and control groups with respect to range of serum vitamin D levels

In the case group, 29 children had serum 25 (OH) vitamin D levels in deficiency range (<20 ng/mL), and only one had level in sufficient range (>20 ng/mL) (Table 2). Age, sex, deft score, and serum 25(OH) vitamin D levels in the control group were collected. In the control group, 13 children had serum 25(OH) vitamin D levels in deficiency range (<20 ng/mL), while 17 had level in sufficient range (>20 ng/mL) (Table 3 and Graph 1 Comparison of the vitamin D levels between case and control groups was done. The comparison of mean vitamin D levels between case and control groups was also done. The case group has a mean serum 25(OH) vitamin D level of 12.19 ng/mL (4.37 SD, 95% confidence interval of 10.5-13.8) and the control group has a mean serum 25(OH) vitamin D level of 20.11 ng/mL (4.12 SD, 95% confidence interval of 18.56-21.65) (Table 4).

**Graph 2:**
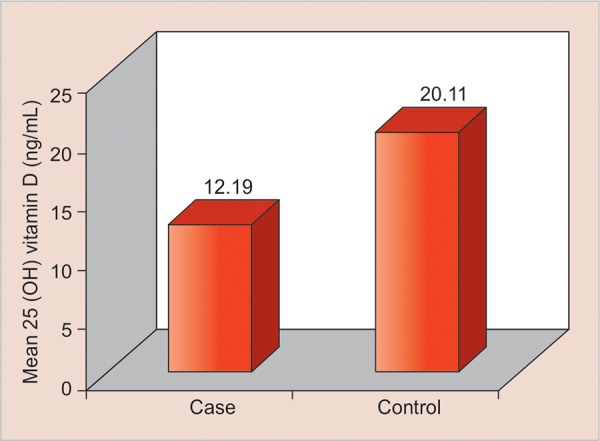
Mean vitamin D levels in case and control groups

The mean levels of serum 25(OH) vitamin D levels were compared between case and control groups and there was a statistically significant difference with a p-value of <0.0001 (t-value of 7.2005) (Graph 2).^2^ Association of vitamin D deficiency and SECC was examined. There was no particular trend of vitamin D level in children with no caries.

**Graph 3:**
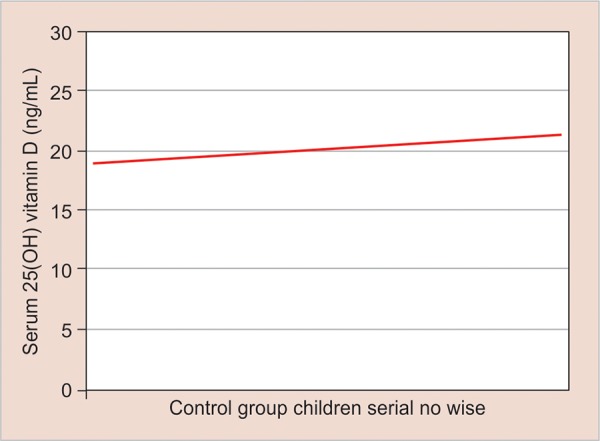
Vitamin D levels in the control group

**Graph 4:**
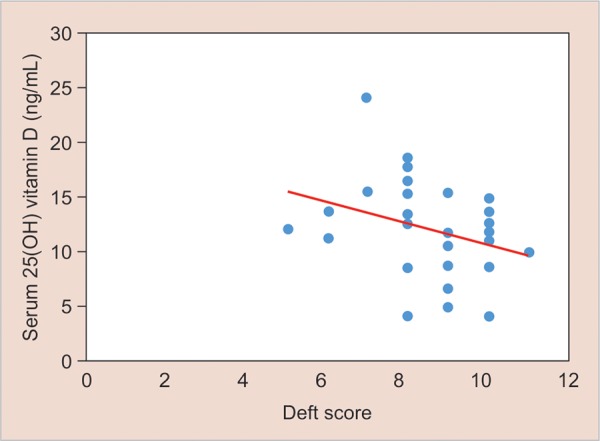
Vitamin D levels in the case group showing correlation between levels of vitamin D and deft score

This is seen as a trait and almost parallel trend line of vitamin D levels plotted with deft score of 0 in each control group child (Graph 3). The correlation between levels of vitamin D and deft score was analyzed by using simple linear regression. The result shows statistically significant inverse correlation between vitamin D levels and SECC (p-value < 0.0001) (Graph 4).

**Table 1:** **Table 1:** Comparison of the demographic data of case and control groups

*Parameter*		*Case group (n = 30) (mean ± SD)*		*Control group (n = 30) (mean ± SD)*		*p-value*	
Age (years)		4.4 ± 0.89		4.5 ± 1.1		0.7	
Male/female		20/10		19/11		0.9	
Deft		8.4 ± 1.3		0		0.00	

**Table 2:** **Table 2:** Deft status and vitamin D levels of case group

*Age (years)*		*Sex*		*Deft status*		*Serum 25(OH) vitamin D (ng/dL)*	
5		M		9		11.7	
5		M		8		16.5	
4		M		7		24.2	
3		M		10		11.2	
5		M		8		13.9	
4		M		11		10	
5		M		9		6.7	
6		M		10		4.2	
5		M		9		5.1	
5		M		10		8.6	
3		F		5		12.1	
4		F		7		15.6	
5		M		6		11.4	
4		F		8		17.8	
5		M		10		12.7	
5		M		8		15.4	
4		F		8		4.2	
3		M		9		10.6	
4		M		10		13.8	
3		M		8		8.4	
4		F		8		13.4	
5		M		8		18.6	
3		F		9		15.4	
5		F		6		13.8	
5		F		10		11.9	
5		M		9		8.7	
6		M		8		12.7	
4		M		10		14.9	
4		F		8		8.7	
6		M		8		13.7	

**Table 3:** **Table 3:** Deft status and vitamin D levels in control group

*Age (years)*		*Sex*		*Deft status*		*Serum 25(OH) vitamin D (ng/dL)*	
5		F		0		21.8	
4		M		0		23.4	
4		M		0		21.9	
3		F		0		20.8	
5		F		0		22	
4		F		0		24.3	
5		M		0		20.3	
3		M		0		15.4	
3		M		0		11.4	
5		F		0		13.6	
6		F		0		8.6	
6		M		0		18.6	
4		M		0		18.4	
5		M		0		21.9	
4		F		0		19.9	
6		M		0		20.1	
5		F		0		23.3	
6		M		0		18.7	
3		M		0		14.5	
6		M		0		24.7	
6		M		0		18.7	
6		M		0		26.7	
6		F		0		24.9	
3		M		0		19.3	
4		M		0		23.6	
4		M		0		20.4	
4		M		0		24.5	
5		F		0		22.9	
4		F		0		19.8	
3		M		0		18.9	

**Table 4:** **Table 4:** Comparison of mean vitamin D levels between case and control groups

*Group*		*Mean vitamin D levels (ng/mL)*		*SD*		*p-value*	
Case		12.19		4.37		<0.0001	
Control		20.11		4.12			

## DISCUSSION

Vitamin D plays a crucial role in oral health by the means of various mechanisms.

Mellanby and Pattison^27^ were the first investigators to find a correlation between vitamin D and dental caries. Vitamin D helps in the absorption of calcium and phosphorus from the intestine^11^ and both calcium and phosphorus help in the mineralization of the teeth.^12^ Vitamin D deficiency *in utero* is found to be associated with enamel hypoplasia because of the metabolic insult to ameloblasts.^28^ Enamel hypoplasia results from defective amelogenesis and is clinically diagnosed by the absence of enamel and by pitting, grooves, or irregularities of enamel.

These defects increase the risk of early colonization by cariogenic bacteria, resulting in caries.^29^ Vitamin D also has an immunological function. It induces the expression of antibacterial proteins like cathelicidin and defensins.^20^ The defensins and cathelicidin have multiple antimicrobial activity against gram-negative and gram-positive bacteria and *Candida albicans* and are effective *in vitro* against oral microorganisms, such as *Streptococcus mutans, Porphyromo-nas gingivalis,* and *Actinobacillus actinomycetemcomitans.^2^*Vitamin D prevents infection by regulating B-cell proliferation and immunoglobulin production.^27^ Vitamin D is essential for the maintenance and utilization of a specific pool of calcium required for normal fluid and electrolyte of the saliva of the parotid gland.^30^ Low salivary flow rate and high viscosity are associated with high dental caries risk.^24^ Various caries-protective factors, such as calcium, inorganic phosphate, pH-increasing substances, and antimicrobial agents are present in saliva.^31^ Hence, vitamin D deficiency is an important environmental factor in predisposition to dental caries.

Vitamin D deficiency is defined as serum levels of 25(OH)D less than 20 ng/dL.^14^ It is considered to be the most common nutritional deficiency.^32^ Though majority of the population in India lives in areas that receive adequate sunlight throughout the year, vitamin D deficiency is nevertheless highly prevalent in all the age groups and both the genders across the country.^33,34^ The prevalence of vitamin D deficiency is 50 to 90% in the Indian subcontinent and is due to low dietary calcium along with dark skin color and indoor lifestyle.^33^ Vitamin D deficiency is due to several reasons like low dietary intake, decreased cutaneous synthesis (because of social and religion-based practice of burkha, seasonal changes , fear of cancer in sunlight, and keeping the child indoor, and dark pigmentation of the skin), high rate of exclusive breast feeding, and deficiency of vitamin D in mother.^35^ In this study, we found statistically significant difference between mean levels of vitamin D in case and control groups.

In the whole sample of both case and control groups, 70% children were deficient in vitamin D and the prevalence of vitamin D deficiency (levels <20 ng/mL) in SECC children was found to be 97%. Brown et al^23^ also determined the prevalence of vitamin D deficiency in children with dental caries and confirmed that a high proportion of children below 5 years, presenting with dental caries, were deficient in vitamin D.

In our study, the prevalence was more, but this high prevalence could be due to the fact that majority of other studies were done in Caucasian population with fair skin and more conversion of cholesterol into vitamin D in skin in contrast to dark skin individuals (Indian) where decreased conversion takes place. Schroth et al^24^ did a pilot study comparing the difference in vitamin D levels in children with SECC and without SECC.

The overall prevalence of vitamin D deficiency was 84%. This difference was statistically significant. Children with SECC had lower concentrations of 25(OH)D (52.9 ± 15.1 *vs* 64.4 ± 21.3 nmol/L, p = 0.032) and were at twice the odds of having inadequate levels (<75 nmol/L). Our study showed the respective value as 12.19 ng/mL in SECC *vs* 20.11 ng/mL in caries-free control.

Schroth et al^25^ multiple regression analysis showed that SECC, low milk consumption, and winter season were significantly associated with lower 25(OH)D concentrations. In this study, significant correlation between vitamin D levels and SECC (p-value 0.0001) is also found. This means that children with lower values of serum vitamin D have more severe dental caries as assessed by deft score.

Hence, vitamin D levels are negatively associated with dental caries. Literature searched has shown that there are studies showing a strong association of low vitamin D levels and dental caries severity, while some studies have not shown any correlation. Elevated serum vitamin D level were associated with better dental health parameters.^36^ Schroth et al^37^ analyzed the relation between vitamin D status and dental caries and found that there exists an association between caries and lower serum vitamin D.

Zhan et al^38^ regarding the association between the concentration of serum vitamin D and incidence of tooth loss and caries status showed that serum vitamin D levels were inversely associated with incidence of tooth loss. Dudding et al^26^ found no evidence of inverse casual effect of vitamin D on dental caries, but they found the association between low vitamin D and early caries onset.

Herzog et al^39^ found no significant association between vitamin D levels and caries experience. In this study, there is statistically significant difference between mean vitamin D levels of children with SECC and children with no caries. Significant correlation between vitamin D levels and SECC was also found. However, this study had certain limitations.

This was a single-center study with small sample size. Therefore, there is a need for large multicenter studies. But our study, which is the first of its kind in India, can be a data base for future studies. Another limitation is that our study was a cross-sectional study and therefore, prospective studies would be needed to see the beneficial effect of vitamin D supplements in decreasing the incidence and severity of dental caries in children.

## CONCLUSION

Dental caries is an important health issue in children affecting their growth, development and quality of life. There are some novel risk factors which are being identified like vitamin D deficiency which predispose to dental caries by multiple mechanisms. There are case-control studies to determine the association between vitamin D levels and dental caries in children, but none is done in India, though vitamin D deficiency is highly prevalent in Indian population.

Our results showed that vitamin D deficiency is an important risk factor both for incidence of dental caries and for its severity in children. In India where despite adequate sunlight vitamin D deficiency is highly prevalent, this is an important modifiable risk factor for dental caries in children. Hence, by supplementing vitamin D in children and preventing the deficiency of vitamin D, dental caries can be prevented.

This will lead to a decrease in the burden of dental caries and help in normal growth and development of children in India.

## References

[1] (2014). Policy on Early Childhood Caries (ECC): classifications, consequences, and preventive strategies. AAPD Reference Manual.

[2] Lal S, Paul D, Vashisht BM (2004). National oral health care programme (NOHCP) implementation strategies. Indian J Community Med.

[3] Damle SG., Damle SG (2012). Epidemiology of dental caries.. Textbook of pediatric dentistry.

[4] Anita FE, Tellis P, Mehta FS (1962). Dental caries experience of school going children in the city of Bombay. J All India Dent Assoc.

[5] Zafar S, Harnekar SY, Siddiqi A (2009). Early childhood caries: etiology, clinical consideration consequences and management. Int Dent SA.

[6] Golak H, Dulgergil CT, Dalli M, Hamidi MM (2013). Early childhood caries update: a review of causes, diagnoses, and treatments. J Nat Sci Biol Med.

[7] Ozdemir D (2013). Dental caries: the most common disease worldwide and preventive strategies. Int J Biol.

[8] Moynihan P, Petersen PE (2004). Diet, nutrition and the prevention of the dental diseases. Public Health Nutr.

[9] Holick MF (2006). High prevalence of vitamin D inadequacy and implication for health. Mayo Clin Proc.

[10] Bischoff-Ferrari HA, Giovannucci E, Willett WC, Dietrich T, Dawson-Hughes B (2006). Estimation of the optimal serum concentration of 25-hydroxyvitamin D for multiple health outcomes. Am J Clin Nutr.

[11] DeLuca HF (2004). Overview of general physiologic features and features and functions of vitamin D. Am J Clin Nutr.

[12] Sobel AE, Hanck A (1948). Calcification of teeth; composition in relation to blood and diet. J Biol Chem.

[13] Grant WB, Holick MF (2005). Benefits and requirements of vitamin D for optimal health: a review. Altern Med Rev.

[14] Holick MF, Binkley NC, Bischoff-Ferrari HA, Gordon CM, Hanley DA, Heaney RP, Murad MH, Weaver CM (2011). Endocrine Society. Evaluation, treatment and prevention of vitamin D deficiency: an Endocrine Society clinical practice guideline. J Clin Endocrinol Metab.

[15] Wortsman J, Matsuoka LY, Chen TC, Lu Z, Holick MF (2000). Decreased bioavailability of vitamin D in obesity. Am J Clin Nutr.

[16] Clemens TL, Adams JS, Henderson SL, Holick MF (1982). Increased skin pigment reduces the capacity of the skin to synthesis vitamin D3. Lancet.

[17] Chen TC, Chimeh F, Lu Z, Mathieu J, Person KS, Zhang A, Kohn N, Martinello S, Berkowitz R, Holick MF (2007). Factors that influence the cutaneous synthesis and dietary sources of vitamin D. Arch Biochem Biophys.

[18] Zhou C, Assem M, Tay JC, Watkins PB, Blumberg B, Schuetz EG, Thummel KE (2006). Steroid and xenobiotic receptor and vitamin D receptor crosstalk mediates CYP24 expression and drug-induced osteomalacia. J Clin Invest.

[19] Grant WB (2011). A review of the role of solar ultraviolet-B irradiance and vitamin D in reducing risk of dental caries. Dermatoen- docrinol.

[20] Hewison M, Vitamin D (2010). The Immune System: new perspectives on an old theme. Endocrinol Metab Clin North Am.

[21] Tao R, Jurevic RJ, Coulton KK, Tsutsui MT, Roberts MC, Kimball JR, Wells N, Berndt J, Dale BA (2005). Salivary antimicrobial peptide expression and dental caries experience in children. Antimicrob Agents Chemother.

[22] Gopinath VK, Arzreanne AR (2006). Saliva as a diagnostic tool for the assessment of dental caries. Arch Orofac Sci.

[23] Brown T, Creed S, Alexender S, Barnard K, Bridges N, Hancock M (2012). Vitamin D deficiency in children with dental caries- a prevalence study. Arch DIS Child.

[24] Schroth RJ, Jeal NS, Kliewer E, Sellers EA (2012). The relationship between vitamin D and severe early childhood caries: a pilot study. Int J Vitam Nutr Res.

[25] Schroth RJ, Levi JA, Sellers EA, Friel J, Kliewer E, Moffatt ME (2013). Vitamin D status of children with severe early childhood caries: a case-control study. BMC Pediatr.

[26] Dudding T, Thomas SJ, Duncan K, Lawlor DA, Timpson NJ (2015). Re-examining the association between vitamin D and childhood caries. PLoS One.

[27] Mellanby M, Pattison CL (1928). The action of the vitamin D in preventing the spread and promoting the arrest of caries in children. Br Med J.

[28] Schroth RJ, Lavelle C, Tate R, Bruce S, Billings RJ, Moffatt ME (2014). Prenatal vitamin D and dental caries in infants. Pediatrics.

[29] Caufield PW, Li Y, Bromage TG (2012). Hypoplasia-associated severe early childhood caries—a proposed definition. J Dent Res.

[30] Dietrich T, Nunn M, Dawson-Hughes B, Bischoff-Ferrari HA (2005). Association between serum concentration of 25-hydroxyvi-tamin D and gingival inflammation. Am J Clin Nutr.

[31] Lagerlof F, Oliveby A (1994). Caries-protective factors in saliva. Adv Dent Res.

[32] Holick MF (2007). Vitamin D deficiency. N Engl J Med.

[33] Harinarayan CV, Joshi SR (2009). Vitamin D status in India—its implications and remedial measures. J Assoc Physicians India.

[34] Marwaha RK, Sripathy G (2008). Vitamin D & bone mineral density of healthy school children in northern India. Indian J Med Res.

[35] Gordon CM, Williams AL, Feldman HA, May J, Sinclair L, Vasquez A, Cox JE (2008). Treatment of hypovitaminosis D in infants and toddlers. J Clin Endocrinol Metab.

[36] Kuhnisch J, Thiering E, Kratzsch J, Heinrich-Weltzien R, Hickel R, Heinrich J (2015). GINIplus study group; LISAplus study group. Elevated serum 25(OH)-vitamin D levels are negatively correlated with molar-incisor hypomineralization. J Dent Res.

[37] Schroth RJ, Rabbani R, Loewen G, Moffatt ME, Vitamin D (2016). dental caries in children. J Dent Res.

[38] Zhan Y, Samietz S, Holtfreter B, Hannemann A, Meisel P, Nauck M, Volzke H, Wallaschofski H, Dietrich T, Kocher T (2014). Prospective study of serum 25-hydroxy vitamin D and tooth loss. J Dent Res.

[39] Herzog K, Scott JM, Hujoel P, Seminario AL (2016). Association of vitamin D and dental caries in children: findings from the National Health and Nutrition Examination Survey, 2005-2006. J Am Dent Assoc.

